# Abstract of the Proceedings of the Buffalo Medical Association

**Published:** 1875-08

**Authors:** Joseph Fowler

**Affiliations:** Sec’y, pro tem.


ART. III.—*Abstract of the Proceedings of the Buffalo Medical Association.* Reported by JOSEPH FOWLER, M. D., Sec'y, *pro tem.*

TUESDAY EVENING, July 6, 1875.

The Association met at their rooms in the Medical College, at the usual hour.

The President, Dr. GOULD, in the chair.

Members present: Drs. Gould, Rochester, Wetmore, Howe, McNiel, Boysen, W. W. Miner, T. M. Johnson, Daggett, Abbot, Samo, Van Peyma, Shannon, Wyckoff, Fowler and Drs. W. H. Johnson, Dorr and Mynter upon invitation.

On motion of Dr. Rochester, Dr. Fowler was appointed Secretary *pro tem.*

Reading of minutes of last meeting dispensed with.

The application for membership of Dr. Herman Mynter was received, and, on motion, it was referred for one month in accordance with the usual regulations, and the doctor invited to participate in the proceedings of the meetings until such time as was necessary to qualify for membership.

The committee appointed at the last meeting to investigate the charges preferred against Dr. J. J. Walsh, submitted a report.

Dr. Rochester moved that the report be accepted. Adopted.

Dr. Hopkins moved that the usual order of business be suspended for the purpose of taking up the matter contained in the report and disposing of it. Carried.

In the absence of Dr. Lothrop, chairman of the committee of investigation, Dr. Wetmore moved that Dr. Hopkins officiate as chairman of said committee, and read the evidence taken in the investigation of the charges preferred against Dr. Walsh. Adopted.

After Dr. Hopkins concluded reading such evidence, the Association proceeded to ballot either for expulsion, suspension or reprimand.

The Chair appointed as tellers Drs. Rochester and Boysen.

The result of the vote was as follows:

For expulsion, 10,
For suspension, 2,
For reprimand, 2.

The President then declared Dr. John J. Walsh expelled from the Association.

Dr. Howe then read a paper on the *Influence of Various Occupations upon the Eye*,* and ended by presenting two illustrative cases, the first was an expample of the accidents frequent among those who manufacture steel instruments or use them in cutting metals. The patient was a boiler maker. Ten weeks before was

*See Art. I. Pg. I.

struck by a small chip from his chisel upon the left eye, a contused wound of the conjuctiva was produced at the inner side of the cornea one-quarter of an inch from its margin. Since then his right has been gradually failing. An examination shows a widely dilated pupil through which flocculent bodies could be readily seen floating in the vitreous, after each motion of the eye. These were portions of the retina detached by the blow together with coagula which might be partially absorbed in time.

The other case was one familliar to Buffalonians generally. He was the negro beggar, who, for the last seven years had plied his vocation at the corner of Niagara and Main streets. Formerly he had been a whitewasher, a class of persons whose eyes are continually exposed to the drops of falling lime. He had met with such an accident. As a result of the repeated irritation an inflammatory process was in both eyes had been set up in the conjunctiva and cornea which was transmitted to the iris, leucomatta covering both pupils, and extensive posterior synechia followed. In spite of the latter complication Dr. Howe, with Dr. Abbot, performed an irodectomy, giving the patient vision sufficiently acute to enable him to pursue any ordinary occupation without difficulty.

Dr. H. R. Hopkins said the Association was under great obligation to Dr. Howe for the address, which was both instructive and suggestive; that the doctor had given us information upon a class of diseases which the profession at large were quite as much interested in as the specialist.

That the subject as treated was peculiarly interesting, from the fact that the text-books usually found in our offices, have little or nothing to say upon the matter, and again as being a contribution to the literature of "preventive medicine," which was of such prominent importance that nothing connected with it could avoid prominence, and this reminded him of what he wanted to say.

That in digging the tunnel for the water works under the Niagara river, the workmen were nearly disabled by a conjunctivitis caused by the sulphurous vapor emitted from certain crevices in rock. That this was of so serious a matter, and so much of an obstacle that the contractors offered thousands of dollars for any means which would relieve the men from the disease.

Dr. ROCHESTER asked what evidence there was that the ophthalmia was dependent on the sulphurous vapor.

Dr. HOPKINS replied that the disease did not appear till the seams emitting this vapor were encountered, and the continued existence of the ophthalmia while the sulphurous vapor was present.

On motion of Dr. WYCKOFF the thanks of the Association were extended to Dr. Howe for his interesting address.

The President oppointed Dr. Abbott as essayist for the next meeting.

Prevailing Diseases—A mild form measles.

